# Genes Encoding GABA-β and HT1D Receptors in Bipolar I (Manic Phase) Patients

**DOI:** 10.29252/NIRP.BCN.9.2.129

**Published:** 2018

**Authors:** Mohammadreza Moradi, Massoud Saidijam, Reza Yadegarazari, Leila Jahangard, Maryam Seifi, Nasrin Matinnia, Ali Ghaleiha

**Affiliations:** 1. Molecular Medicine Research Center, Hamadan University of Medical Sciences, Hamadan, Iran.; 2. Behavioral Disorders and Substance Abuse Research Center, Hamadan University of Medical Sciences, Hamadan, Iran.; 3. Department of Nursing, Faculty of Science, Hamadan Branch, Islamic Azad University, Hamadan, Iran.

**Keywords:** GABA-β3, HT1D, Serotonin, Bipolar disorder, Gene expression

## Abstract

**Introduction:**

According to the cumulative evidence, genes encoding GABA receptors inhibit neurotransmitters in CNS and are intricately involved in the pathogenesis of mood disorders. Based on this hypothesis, these genes may be expressed in bipolar patients. As a result, we evaluated the gene expressions of GABA-β3 and HT1D receptors to assess their associations with bipolar mood disorder.

**Methods:**

In this study, 22 patients with bipolar I disorder (single manic episode) and 22 healthy individuals were enrolled. All participants were older than 15 years and had referred to Farshchian Hospital, Hamadan, Iran. They were diagnosed based on DSM IV–TR criteria and young mania rating scale in order to determine the severity of mania by a psychiatrist as bipolar Type 1 disorder in manic episode. We evaluated the expression of GABA–β3 and HT1D receptor genes in peripheral blood mononuclear cells, using real-time RT-PCR analysis.

**Results:**

In our study, a reduction in the gene expression of GABA–β3 and HT1D receptors was observed in peripheral blood mononuclear cells of the patients with bipolar disorders compared to the healthy controls.

**Conclusion:**

The results of this study supports the hypothesis that the gene expression for serotonin and GABA receptors can be employed in elucidating the pathogenesis of bipolar disorders.

## 1. Introduction

Several compounds have been identified that affect 5-HT system (e.g. selective serotonin reuptake inhibitors) and are used in the treatment of bipolar depression (BP). For this reason, the genes of the serotonin system have been considered as good candidates for BP and been investigated in several studies (Mundo, Zai, Lee, Parikh, & Kennedy, 2001). The specific roles of GABAergic neurons in the pathogenesis of these diseases are not well understood. GABA is a one of the major neurotransmitters whose expression starts from the embryonic stage and continues throughout life. At an early developmental stage, this major neurotransmitter acts as an excitatory factor and has been implicated in several processes, including neurogenesis (Wu & Sun, 2015).

γ-Amino Butyric Acid (GABA) is the main inhibitory neurotransmitter in the brain and controls many processes during the brain development. Approximately 20% of all central nervous system neurons are GABAergic (Somogyi, Tamas, Lujan, & Buhl, 1998). It has been hypothesized that the hypoactivity of the GABAergic signaling system contributes to the pathologies of schizophrenia, bipolar disorder, and major depressive disorder (Charych, Liu, Moss, & Brandon, 2009; Fatemi, Folsom, Rooney, & Thuras, 2013; Gonzalez-Burgos, Fish, & Lewis, 2011; Luscher, Shen, & Sahir, 2010; Mohler, 2012).

Serotonin (5-hydroxytryptamine; 5-HT) is another key neurotransmitter in the central nervous system. Presumably, dysfunction of the serotonergic system can result in several psychiatric diseases such as affective and anxiety disorders. This neurotransmitter exerts its various effects through multiple receptors, therefore serotonin receptors (HTRs) are the subject of comprehensive research on the pathophysiology of many mental diseases (Cruceanu, Alda, Rouleau, & Turecki, 2011). There is increasing evidence that genes encoding GABA and HTR1D receptors are inhibitory neurotransmitters in CNS that are involved in the pathogenesis of mood disorders. To determine whether or not the increasing GABA–β3 and HT1D receptors are engaged in the etiology of bipolar disorder, we enrolled 22 patients with bipolar I disorder (single manic episode) and 22 healthy controls in a case-control study. Furthermore, we assessed the expression of HTR1D and GABA-β3 receptor genes in their peripheral blood mononuclear cells. To the best of our knowledge, this study is the first research focusing on the gene expressions of GABA–β3 and HT1D receptors in peripheral blood mononuclear cells.

## 2. Methods

### 2.1. Clinical sample collection

In the present case-control study, we enrolled 22 patients and 22 healthy controls. All participants were over 15 years old who had referred to Farshchian Hospital, Hamadan, Iran. In this study, 22 patients with bipolar I disorder (single manic episode) were diagnosed based on DSM IV–TR criteria and were interviewed by a psychiatrist. Young Mania Rating scale was used to determine the severity of mania level. The patients who had other major psychiatric problems, including medical disorders, a history of these diseases and substance abuse, and alcohol or drug consumption, were excluded. The selection of the controller, sex, and age of the patients and the controls was exactly adapted.

We also utilized a predetermined questionnaire consisting of demographic characteristics; age, sex, exposure to carcinogens, smoking habits, and residence. Our study protocol was approved by the Ethics Committee of Hamadan University of Medical Sciences and Health (Hamadan, Iran). Subsequently, the study objectives were explained to the patients, and written informed consent was taken from them. Table 1 presents some demographic characteristics of case and control groups.

**Table 1.** Some demographic characteristics of case and control group

	**Sex**		**Mean Age, y**	**Family History**		**Drug History**		**Smoking Habits**		**Residence**	

	**Male**	**Female**		**Yes**	**No**	**Yes**	**No**	**Yes**	**No**	**Urban**	**Rural**
Case	11	11	32		22		22		22	11	11
Control	11	11	32		22		22		22	11	11

### 2.2. Separation of mononuclear cells

Blood samples from all subjects were taken at 8:00 AM each day. Two milliliter of blood was taken from each participant, and the mononuclear cells were separated according to Denis English and Burton R. Andersen (1974) method. The heparinized human blood samples were diluted with an equal volume of saline and layered on the gradient. The tubes were centrifuged at 800 g for 20 min at 4°C. After the samples were centrifuged, three distinct cell layers were observed: the first layer was plasma solution interface which consisted of mononuclear cells and platelets, the second layer was solution I-II interface which consisted of polymorphonuclear cells, and the third layer was erythrocytes. The separation was carried out in accordance with Denis English and Burton R. Andersen method (English & Andersen, 1974).

### 2.3. Total RNA extraction

Total RNA was isolated from the samples by applying RNeasy Mini Kit (Qiagen Inc., USA) according to the manufacturer’s protocol. The extracted RNA was dissolved in 40 μL RNase- free water, depending on the quantity of the precipitation. The RNA concentration and its purity were performed by a NanoDrop spectrophotometer (Bio-TeK, USA). Determination of integrity and the quality of RNA were evaluated by 1% agarose gel electrophoresis.

### 2.4. Primers designing

All primers were designed by Allel ID 6 (PRIMER Biosoft Co. USA), considering variants of each gene. Due to the high stability of 18S rRNA as a housekeeping gene, it was regarded as a normalizer gene in this study (Bas, Forsberg, Hammarström, & Hammarström, 2004). Table 2 presents the specifics of the primers.

**Table 2.** The specifics of designed primers for HTR1DR, GABRβ3, and 18srRNA

**Property**	**HTR1DR**	**GABRβ3**	**18s rRNA**
NCBI accession number	NM_000864	NM_021912	X03205
Forward primer	ATCAGCATCGCCTATACCATCAC	CAGTGCTGTATGGGCTCAGAATCA	GTAACCCGTTGAACCCCATT
Primer length	23	24	20
Amount of use	10 Picomol	10 Picomol	10 Picomol
Reverse primer	TCCAGAGCAATGACACAGAGATG	AACTCAATGTCATCCGTGGTGTAGC	CCATCCAATCGGTAGTAGCG
Primer length	23	25	20
Amount of use	10 Picomole	10 Picomole	10 Picomole
Amplicon length	125	130	152
Optimized annealing temperature	62	47.5	53.5

### 2.5. cDNA synthesis

Quanti Test Reverse Transcription kit (Qiagen Inc., USA) was employed for cDNA synthesis. There was a solution for decomposing of genomic DNA in each kit. According to the manufacturer’s protocol, 12 μL of the extracted RNA and 2 μL of the decomposed genomic DNA solution were mixed and incubated at 42ºC for 2 min. This mixture was then mixed with the main components containing 1 μL of reverse transcriptase enzyme, 1 μL of primer, and 4 μL of buffer. This final mixture was incubated at 42ºC for 15 s and then at 95ºC for 3 min in order to complete the cDNA synthesis stage. All heating steps were conducted in a conventional PCR.

### 2.6. Real-time RT-PCR analysis

In order to determine the mRNA expression, we applied the Quanti Fast SYBER green PCR kit (Qiagen Inc., USA). All components required in this kit are the same as those in “master-mix solution.” The main components for real-time RT-PCR analysis were 100 ng of template, 10 μL of master mix, 10 pmol of primer, and 10 μL of distillated water. Real-time RT-PCR analysis was performed under the following conditions presented in Table 3. The results were quantified using a CFX96 real-time PCR detection system (Bio-Rad, USA).

**Table 3.** Temperature and time values in Real-time RT-PCR analysis

**Real-time Step**	**Temperature**	**Duration**
Initial activation	95°C	5 min
40 cycles of:		
Denaturation	95°C	15 s
Annealing	Optimized annealing temperature	30 s
Extension	72°C	30 s

### 2.7. Statistical analysis

The statistical analysis was performed using SPSS-16 (SPSS Inc., USA). All values were reported as mean±SD. The result of Kolmogorov-Smirnov test showed the normal distribution of the data (P=0.132). We employed t test to compare the gene expressions of GABA–β3 and HT1D receptors in peripheral blood mononuclear cells of bipolar I (manic phase) patients and normal subjects. P value less than 0.05 was considered significant.

## 3. Results

**Figure 1.**
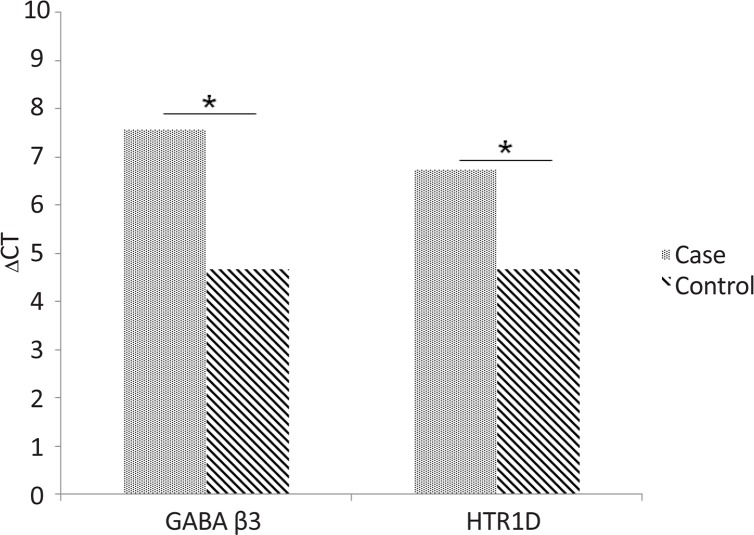
Gene expressions of GABA–β3 and HT1D receptors was observed in peripheral blood mononuclear cells of the patients with bipolar disorders compared to the healthy controls (P<0.05).

In our study, a reduction in the gene expressions of GABA–β3 and HT1D receptors was observed in peripheral blood mononuclear cells of the patients with bipolar disorders compared to the healthy controls (P=0.005 and 0.008, respectively). Table 4 presents the results which are displayed as mean and standard deviation. P value less than 0.05 was considered significant. Figure 1 shows the result of t test. P value less than 0.05 was considered significant.

**Table 4.** ΔCT values of GABA –β3 and HT1D receptor gene in patients and normal subjects

		**Control**		**Cases**		**Difference**		**Paired t Test**	
**Variable**	**Samples**	**Mean**	**SD**	**Mean**	**SD**	**Mean**	**SD**	**P**
GABA β3	22	4.67	1.22	7.58	2.08	3.18	−0.86	0.005
HTR1D	22	4.23	1.74	6.75	1.68	2.52	−0.06	0.008

## 4. Discussion

Bipolar disorder is a brain and neuropsychiatric disorder, leading to unusual changes in patients’ behavior, energy level, cognition, and ability to perform daily activities. The symptoms begin in the late teens; however, in some patients, the signs and symptoms are observed in their childhood or the following decades. The symptoms occur in a period called episode. This period includes an assault course with or without depression. Bipolar disorder (BP) is an intense mental disorder distressing approximately 1% of the world’s population and is distinguished by mood swings from elation to depression. The hereditary factors have been proved to be associated with BP, although the etiology of this disorder remains unknown. According to Mitchell (1993), Kraepelin was the first researcher who showed a congenital link in about 80% of cases and a central role of genetic factors in BP. The results obtained from family, twins, and adoption studies also indicate the presence of a strong hereditary component; moreover, the advances in molecular genetics since the 1960s support these epidemiological findings (Barnett & Smoller, 2009).

Since 2000, with the completion of the Human Genome Project, the number of molecular genetic studies has greatly increased, and many chromosomal regions have been implicated in BP. Nevertheless, no single susceptible gene has been identified, and the findings have not been replicated (Arisoy & Oral, 2009). However, a majority of the findings of molecular genetics, pharmacokinetics, and pharmacodynamics about major depression have been conducted without the positive replication in independent samples. This might be due to small samples, race-related stratification, dissimilarity in mean age, or comorbidities with either mental or somatic disorders across different studies. Another reason accounting for the unrepeatable genetic studies in depression is the heterogeneous clinical phenotype in patients with major depression in these studies (Domschke, 2013).

There are a few studies focusing on gene expression in bipolar patients, especially those with bipolar I, because there is no easy access to patients who have not received any drugs. Most of these studies have been done after the patients’ death and this is another reason for the low number of these studies. We believe that our research is valuable because a non-invasive method has been applied in this study. We examined the gene expressions of GABA–β3 and HT1D receptors in peripheral blood mononuclear cells. Gene expression in bipolar patients, particularly in various tissues except for the central nervous system tissue, has recently been studied. Thus far, a few genes have been investigated and their effectiveness has been proved. Our findings revealed a significant decrease (82%) in gene expressions of GABA–β3 and HTR1D receptors in bipolar patients who did not receive any drugs compared to the healthy controls (P<0.001).

Better understanding of gene expressions of these receptors can contribute to the efficient use of agonist and antagonist to treat such diseases. In patients with a familial risk factor, gene expressions can be employed as an indicator to predict the likelihood of disorder along with the changes in episodic phases of the disorder to prevent or treat the disease in its initial stages or the subsequent attacks. To determine the interaction of genes polymorphism and factors such as environmental ones, we recommend that further studies be conducted on the varieties of genes and the final proteins. Moreover, future research should investigate the impacts of gene expression on brain tissues and cerebrospinal fluid and compare these impacts with the results of similar studies on blood samples. It should be noted that this objective necessitates the studies on brain after its death; accordingly, the results of these studies cannot be generalized to live patients and these analyses will have their own limitations. We also recommend that the research on the expression of specific genes be performed before and after the treatment to explore the effects of drugs on gene expressions. It should be noted that the changes in the expression of individual genes in peripheral blood cells alone might not be a reliable biomarker for the diagnosis of mental disorders. There is no doubt that more studies are needed to confirm our results.

In the present study, we observed a significant reduction in gene expressions of GABA–β3 and HT1D receptors in peripheral blood mononuclear cells of patients with bipolar disorders compared to healthy controls. The strength of our investigation lies in the use of a non-invasive method. We assessed gene expressions of GABA– β3 and HT1D receptors in peripheral blood mononuclear cells. Our findings suggest that the use of a non-invasive method focusing on gene expression provides a new strategy for the diagnosis of mental disorders. We believe that detection of gene products (proteins) in peripheral blood products will also potentially generate more reliable results.
